# The Clinicopathological Features and Genetic Mutations in Gastric Cancer Patients According to EMAST and MSI Status

**DOI:** 10.3390/cancers12030551

**Published:** 2020-02-27

**Authors:** Wen-Liang Fang, Ming-Huang Chen, Kuo-Hung Huang, Shih-Ching Chang, Chien-Hsing Lin, Yee Chao, Su-Shun Lo, Anna Fen-Yau Li, Chew-Wun Wu, Yi-Ming Shyr

**Affiliations:** 1 Division of General Surgery, Department of Surgery, Taipei Veterans General Hospital, Taipei City 11217, Taiwan; 2 School of Medicine, National Yang-Ming University, Taipei City 11217, Taiwan; 3 Center of Immuno-Oncology, Department of Oncology, Taipei Veterans General Hospital, Taipei City 11217, Taiwan; 4 Division of Colon & Rectal Surgery, Department of Surgery, Taipei Veterans General Hospital, Taipei City 11217, Taiwan; 5 Genome Research Center, National Yang-Ming University, Taipei City 11217, Taiwan; 6 Department of Surgery, National Yang-Ming University Hospital, Yilan County 26058, Taiwan; 7 Department of Pathology, Taipei Veterans General Hospital, Taipei City 11217, Taiwan

**Keywords:** EMAST, MSI, MSI-H, MSS, gastric cancer, clinicopathological feature, genetic mutation

## Abstract

*Background:* There has been no report regarding the clinicopathological features and genetic mutations regarding elevated microsatellite alterations at selected tetranucleotide repeats (EMAST) in gastric cancer (GC). *Methods:* The correlation among EMAST status, microsatellite instability (MSI) status, mutations of common GC-related genes and 16 DNA repair-associated genes, and the clinicopathological features were analyzed. *Results:* Among the 360 GC patients enrolled, there were 76 (21.1%) with EMAST+ tumors and 284 with EMAST− tumors, and 59 (16.4%) were MSI-high (MSI-H) tumors, and 301 were microsatellite stable (MSS) tumors. Patients with EMAST+ tumors exhibited an earlier pathological T category and had more genetic mutations in the *PI3K*/*AKT* pathway, *ARID1A* and DNA repair-associated genes than those with EMAST− tumors. Patients with MSI-H tumors have more genetic mutations in the *PI3K*/*AKT* pathway and DNA repair-associated genes than those with MSS tumors. In the subgroup analysis for MSI-H GC, EMAST+ tumors were associated with earlier pathological T and N categories, earlier TNM stages, higher frequency of DNA-repair-associated genetic mutations, and a better survival rate than EMAST− tumors. *Conclusions:* *PI3K*/*AKT* pathway mutations may play an important role in EMAST+ and/or MSI-H GC. EMAST+/MSI-H tumors seem to represent a different subtype of gastric cancer from EMAST−/MSI-H tumors.

## 1. Introduction

Gastric cancer (GC) ranks as the sixth most common cancer and the second leading cause of cancer-related deaths [1]. According to The Cancer Genome Atlas (TCGA) [2], GC is classified into four types: (1) Epstein–Barr virus (EBV) positive, (2) microsatellite instability-high (MSI-H), (3) genomically stable, and (4) GC with chromosomal instability. Immunotherapy has been shown to have a better disease control rate in GC patients with MSI-H tumors than in those with microsatellite stable (MSS) tumors [3]. Elevated microsatellite alterations at selected tetranucleotide repeats (EMAST), a variant of MSI with a prevalence ranging from 9% to 75% [4], have been reported in various cancers. To date, there has been no report regarding EMAST status in GC.

In colorectal cancer, the incidence of EMAST was similar to that of MSI and was approximately 20%–40%; EMAST+ tumors were associated with the MSI-H phenotype and more frequently located in the colon than in the rectum [5,6]. However, in non-small cell lung cancer [7], the incidence of EMAST was higher than that of MSI (42.9% vs. 16.3%), and there was no association between the incidence rates of EMAST and MSI. The correlation between EMAST status and patient survival in cancer is still controversial [5,6,7,8]. Consequently, there is a need to investigate the correlation among EMAST status, MSI status, genetic alterations, and clinicopathological features in GC patients.

In our previous study [9], we designed a 16 DNA-repair-associated gene panel, using next-generation sequencing (NGS), to investigate the clinical impact of EMAST/MSI status in colorectal cancer. We found that, in MSI-H colorectal cancer, EMAST+ tumors were associated with a better prognosis than EMAST− tumors. In this study, we used a 16-gene panel to study the correlation between the clinicopathological features, the mutation profiling of DNA-repair-associated genes and of common GC-related genes, and the prognosis of GC patients according to the EMAST and MSI status.

## 2. Results

Among the 360 patients, 76 (21.1%) had EMAST+ tumors and 284 had EMAST− tumors; and 59 (16.4%) tumors were MSI-H, and 301 were MSS. According to the EMAST/MSI status, there were 35 EMAST+/MSI-H, 41 EMAST+/MSS, 24 EMAST−/MSS, and 260 EMAST−/MSS tumors.

### 2.1. Clinicopathological Profiles

As shown in Table 1, patients with EMAST+ tumors had fewer Borrmann type 3 and 4 tumors, fewer Helicobacter pylori (HP) infections, earlier pathological T categories, and more genetic mutations in the *PI3K*/*AKT* pathway and in *ARID1A* than those with EMAST− tumors. Low expression of MSH3 by IHC staining was not significantly different between patients with EMAST+ tumors and patients with EMAST− tumors (28.9% vs. 27.8%). Patients with MSI-H tended to be older, have a larger tumor size, have more EBV infections, have fewer HP infections, and have more genetic mutations in the *PI3K*/*AKT* pathway than those with MSS.

As shown in Table 2, patients with EMAST+/MSI-H tumors had an earlier pathological T category, earlier pathological TNM stage, and had fewer Borrmann type 3 and 4 tumors than the other three GC subtypes.

### 2.2. Mutational Profiling of GC Subtypes According to EMAST/MSI Status

Mutation profiling of DNA-repair-associated genes, using NGS analysis, was performed for 160 patients, according to their EMAST/MSI status. As shown in Table 3, EMAST+ tumors were associated with a significantly higher frequency of genetic mutations than EMAST− tumors in *EXO1*, *EPCAM*, *MSH2*, *TGFBR2*, *MLH1*, *MSH3*, *POLE*, *AXIN1*, *AXIN2*, and *BAX*. MSI-H tumors showed a significantly higher frequency of genetic mutations than MSS tumors in *EXO1*, *EPCAM*, *PMS1*, *TGFBR2*, and *BAX*, while MSS tumors showed a significantly higher frequency of genetic mutations than MSI-H tumors in *CTNNB1*.

As shown in Table 4, EMAST+/MSI-H tumors demonstrated a significantly higher frequency of genetic mutations in *EXO1*, *EPCAM*, *MSH2*, *MSH6*, *TGFBR2*, *AXIN1*, and *BAX* than the other three subtypes. EMAST+/MSS tumors showed a significantly higher frequency of genetic mutations in *MSH3* and *POLE* than the other three subtypes.

### 2.3. Initial Recurrence Patterns

Among the 360 patients, 275 patients receiving curative surgery were enrolled in the analysis of initial recurrence patterns. As shown in Table 5, patients with EMAST+ tumors had fewer distant metastases (8.6% vs. 20.7%, *p* = 0.034) than those with EMAST− tumors. There was no significant difference in the initial recurrence pattern between patients with MSI-H tumors and patients with MSS tumors.

As shown in Table 6, patients with EMAST−/MSI-H tumors had significantly more tumor recurrence than the other subtypes (EMAST−/MSI-H: 50%, EMAST+/MSS: 31.0%, EMAST−/MSS: 25.9%, EMAST+/MSI-H: 0%, *p* = 0.001). Among the initial recurrence patterns, patients with EMAST−/MSI-H tumors were associated with the highest distant metastasis rates compared to the other three GC subtypes, especially peritoneal recurrence.

### 2.4. Survival Analysis

The five-year overall survival (OS) rates were not significantly different between patients with EMAST+ and patients with EMAST− tumors (65.5% vs. 60.2%, *p* = 0.689), or between patients with MSI-H and patients with MSS tumors (60.0% vs. 61.6%, *p* = 0.793).

**Figure 1:**
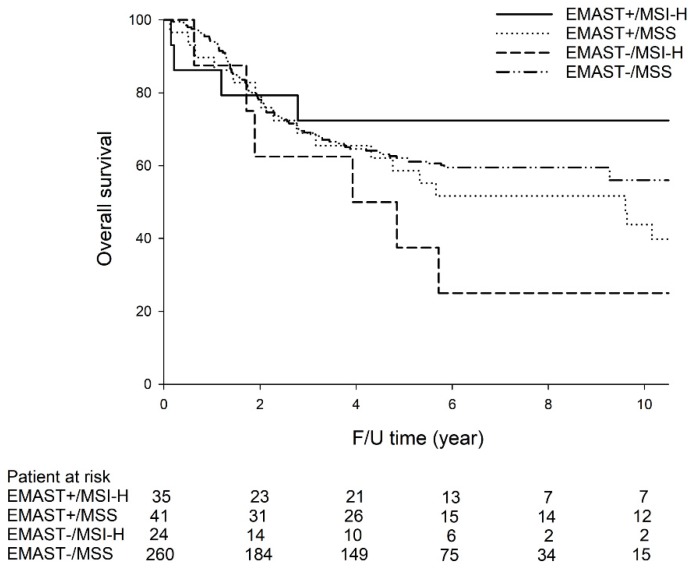
The five-year OS rates (72.4% vs. 37.5%, *p* = 0.046) were significantly higher in GC patients with EMAST+/MSI-H than in GC patients with EMAST−/MSI-H. There was no significant difference in five-year OS rates between other GC subtypes.

As shown in Figure 1, among the 275 patients receiving curative surgery, the five-year OS rates were the highest in patients with EMAST+/MSI-H (72.4%), followed by EMAST−/MSS (62.1%), EMAST+/MSS (58.6%), and EMAST−/MSI-H (37.5%). Among the four GC subtypes, patients with EMAST+/MSI-H had significantly higher five-year OS rates compared with patients with EMAST−/MSI-H tumors (72.4% vs. 37.5%, *p* = 0.046). There was no significant difference in five-year OS rates between other GC subtypes.

As shown in Table 7, multivariate analysis showed that lymphovascular invasion, Lauren’s classification, and pathological TNM stage were independent prognostic factors affecting OS. Multivariate analysis demonstrated that lymphovascular invasion, Lauren’s classification and pathological TNM stage were independent prognostic factors affecting disease-free survival (DFS) (Table 7). 

### 2.5. Subgroup Analysis for MSI-H GC

For clinicopathological features of MSI-H GC, EMAST+ tumors showed fewer Borrmann type 3 and 4 tumors, less lymphovascular invasion, more lymphoid stroma, earlier pathological T and N categories, and earlier TNM stages than EMAST− tumors.

Regarding the mutational profiling in MSI-H GC, EMAST+ tumors showed a significantly higher frequency of genetic mutations in *MSH2*, *AXIN1*, and *POLD1* than EMAST− tumors.

For the initial recurrence pattern and survival analysis for MSI-H GC patients receiving curative surgery, EMAST+ tumors showed less tumor recurrence and a better five-year OS rate than EMAST− tumors.

## 3. Discussion

To the best of our knowledge, this study is the first to investigate the clinical impact of EMAST status on genetic alterations and clinicopathological features in GC. Our results demonstrated that *PI3K*/*AKT* pathway mutations were more frequent in EMAST+ and/or MSI-H tumors. Neither EMAST status nor MSI status was an independent prognostic factor. Subgroup analysis for MSI-H GC showed that EMAST+ tumors were associated with more favorable clinicopathological features and better survival than EMAST− tumors, demonstrating that EMAST+ and EMAST− tumors are different entities in MSI-H GC.

It was reported that EMAST was associated with the loss of MSH3 nuclear expression in colorectal cancer [10], while no significant correlation between EMAST and loss of MSH3 expression was reported in pancreas cancer [11]. Although our results demonstrated that EMAST status was not associated with low expression of MSH3, the frequency of MSH3 mutation in EMAST+/MSI-H and EMAST+/MSS tumors was 23.5% and 27.5%, which was significantly higher than that in EMAST−/MSI-H and EMAST−/MSS tumors (8.3% and 3.8%). The MSH3 mutation might play an important role in EMAST status in GC.

In the present study, regarding the 16 DNA-repair-associated genes, EMAST+/MSI-H tumors had a higher frequency of *EXO1*, *EPCAM*, *MSH2*, *MSH6*, *TGFBR2*, *AXIN1*, and *BAX* than the other three GC subtypes. Our previous study [9] regarding mutations in DNA-repair-associated genes in colorectal cancer demonstrated that EMAST+/MSI-H tumors had a higher frequency of *MLH1*, *MSH3*, *MSH6*, *PMS2*, and *EXO1* genetic mutations than the other three colorectal cancer subtypes. Comparing the results of the present study and our previous study [9] in colorectal cancer, we observed that *MSH6* and *EXO1* genetic mutations were higher in EMAST+/MSI-H tumors than other subtypes, in both GC and colorectal cancer. It seems that *MSH6* and *EXO1* genetic mutations play an important role in gastrointestinal tract cancer with the EMAST+/MSI-H phenotype. Because there have been no reports investigating the differences in DNA-repair-associated genetic mutations among GC patients according to the EMAST/MSI status, our results might provide useful information for future studies in this field. More patients encompassing different races enrolled from different countries and further in vivo and in vitro studies are required to validate our results.

Although immunotherapy was approved by the US Food and Drug Administration for MSI-H tumors, the response rate was approximately 30%–40% [12,13]. The most important finding of the present study is that, for MSI-H GC patients, EMAST+ tumors were associated with more favorable clinicopathological features, a better prognosis, and a higher frequency of genetic mutations in *MSH2*, *AXIN1*, and *POLD1* compared with EMAST− tumors. Consequently, the higher frequency of several DNA-repair-associated genetic mutations in EMAST+/MSI-H than EMAST−/MSI-H tumors demonstrated that combined EMAST/MSI status may be more promising than MSI status alone for the application of immunotherapy in GC treatment, which was similar to the findings of our previous study in colorectal cancer [9]. For validation of our results and hypothesis, more patients enrolled from different countries and clinical trials are required for the application of EMAST/MSI status on the immunotherapy for GC patients.

In the present study, for MSS GC, the status of EMAST does not correlate with patient prognosis. There are two possible reasons. First, the patient number is limited and the difference is not easy to reach statistical significance. Second, in comparison with the major role of MSI status associated with a better prognosis, EMAST phenotype may play as an additional effect on improved prognosis. Only for MSI-H GC, EMAST+ tumors were associated with significantly more DNA-repair-associated genetic mutations than EMAST− tumors, which may cause immune response and improve patient survival.

In previous studies [14,15], Corso G et al demonstrated that MSI-H GC had distinct clinicopathological features and frequently showed activation of *PI3K/AKT* pathway compared with MSS GC, which was similar to our findings (Table 1). Furthermore, one of another important findings is that *PI3K*/*AKT* pathway mutations were more frequent in EMAST+ tumors than in EMAST− tumors, which was also observed in the MSI-H tumors than in MSS tumors (Table 1). Our previous study [9] in colorectal cancer also demonstrated a higher incidence of mutations in *PI3K*/*AKT* pathway genes (*PIK3CA*, *PTEN*, and *AKT1*) in the EMAST+/MSI-H tumors than in other subtypes. It was reported that upregulation of *PI3K/AKT* pathway was observed in tumors with mismatch repair deficiency, including MSH2-mutant tumors [16]. In addition, overexpression of *AXIN1* protected against tumors via inhibiting the PI3K/AKT pathway [17]. We speculate that mutations of *MSH2* and *AXIN1* may be involved in the *PI3K/AKT* pathway and play an important role in both EMAST+ and MSI-H tumors originating from gastrointestinal tract cancer. Further in vivo and in vitro studies are required to investigate the mechanism between EMAST status, MSI status, *MSH2*, *AXIN1*, and *PI3K*/*AKT* pathway in GC. Our findings might have clinical impact on the targeted therapy for EMAST+ and MSI-H GC.

There are some limitations in the present study. First, it is a retrospective study, and selection bias exists. Second, although significant survival difference was observed between the EMAST+/MSI-H group and EMAST−/MSI-H group, the patient number enrolled in the present study was limited, and more patients are required for the validation of our results. We hope our findings can have a clinical impact on immunotherapy and targeted therapy for GC treatment in the future.

## 4. Materials and Methods

### 4.1. Patients and Tissue Collection

The normal and tissue samples of 360 GC patients who underwent curative surgery were obtained from the biobank of our hospital. After surgery, tumor and normal tissues were collected and immediately frozen and stored in liquid nitrogen. The study was approved by the Ethical Committee of Taipei Veterans General Hospital (Number: 2017-12-012CC). The study was performed in accordance with the Declaration of Helsinki. Written informed consent was obtained from all study participants.

The clinical data, including age, gender, tumor location, TNM stage, differentiation, pathological prognostic features, and follow-up conditions, were prospectively collected. After surgery, patients were followed up quarterly for the first 3 years and then semiannually thereafter. The follow-up examinations included panendoscopy, serum tumor markers (CEA, CA19-9), chest radiography, and sonography or computed tomography of the abdomen.

DNA samples were extracted from freshly frozen tumors and normal tissues (surgical resection margins or normal tissues were sampled distant from the primary tumor site), using the QIACUBE (Qiagen, Cat.51306, Hilden, Germany) instrument and dedicated reagents and kits, according to the manufacturer’s instructions.

### 4.2. Analysis of MSI and EMAST Statuses

According to international criteria, five reference microsatellite markers were used to determine MSI status: D5S345, D2S123, BAT25, BAT26, and D17S250. The MSI method was the same as that described in a previous report [18]. Samples with two or more MSI markers were defined as MSI-H, and those with one or no MSI markers were classified as MSS.

As described in a previous study [19], five tetranucleotide microsatellite markers were used to determine EMAST status: (MYCL1, D9S242, D20S85, D8S321, and D20S82). If two or more of the 5 markers showed instability, tumors were defined as EMAST+; if none or one of the markers showed instability, the tumor was considered to be EMAST−.

### 4.3. Identification of HP and EBV Infection

The methods for identifying HP and EBV infection were the same as those described in a previous report [20]. HP infection was identified by polymerase chain reaction (PCR), and EBV infection was detected by using the Sequenom MassARRAY system.

### 4.4. Identification of PIK3CA Amplification

As described in a previous study [21], the copy number of the PIK3CA gene was analyzed by quantitative real-time PCR, and the LINE1 element was used as an internal reference target, using primer sequences. Copy number amplification of the PIK3CA gene was defined by a copy number ≥3 with a *p*-value <0.05.

### 4.5. Mutation Analysis of Common GC-related Genes Based on MassARRAY

As described in a previous study [22], a nine-gene panel using MassARRAY was performed for mutation analysis of common GC-related genes in all 360 GC patients enrolled, including *TP53*, *ARID1A*, *PTEN*, *PIK3CA*, *AKT1*, *AKT2*, *AKT3*, *KRAS*, and *BRAF*. Among them, mutations in the *PI3K*/*AKT* pathway were identified in at least any one of the following genes: *PTEN*, *PIK3CA*, *AKT1*, *AKT2*, and *AKT3*.

### 4.6. Next-Generation Sequencing

As described in our previous study [9], we used the HiSeq2500 system (Illumina Inc., San Diego, CA, USA) to explore the DNA sequences of all exons of 16 well-known DNA-repair-related genes in 150 GC patients, including *AXIN1*, *AXIN2*, *BAX*, *CTNNB1*, *EPCAM*, *EXO1*, *MLH1*, *MSH2*, *MSH3*, *MSH6*, *PCNA*, *PMS1*, *PMS2*, *POLD1*, *POLE*, and *TGFBR2*.

### 4.7. Immunohistochemical Staining for MSH3

Tissue sections of 5 µm thickness were deparaffinized, rehydrated, and pretreated with sodium citrate buffer (10 mM, pH 6.0), in a pressure cooker, at 121 °C, for 3 minutes. Immunohistochemical (IHC) staining was performed with the Novolink Poly Detection System (Cat.RE7280, Leica Biochemistry, Newcastle upon Tyne, UK), according to the manufacturer’s instructions. The tissue sections were incubated at 4 °C overnight, with MSH3 primary antibody (Cat.ab111107, 1:500 dilution, Abcam, Cambridge, UK). The samples were developed with DAB chromogen and then counterstained with hematoxylin. The slides were mounted, using DPX Mountant for histology (Cat.44581, Sigma, Gillingham, UK). As defined in previous reports [8,23], low MSH3 protein expression was defined as <85% brown staining of cell cores in tumor cells, and high MSH3 protein expression was defined as ≥85% brown staining of cell cores in tumor cells.

### 4.8. Statistical Analysis

Statistical analyses were performed by using IBM SPSS version 25.0. Categorical data were compared, using a χ2 test, with Yates correction or Fisher’s exact test. OS was measured from the operation date to the date of death or the final follow-up. DFS was defined as the length of time after surgery during which a patient survived without GC recurrence. The distributions of OS and DFS were estimated, using the Kaplan–Meier method. Multivariate analysis, using Cox proportional hazards models, was performed to explore the association of the clinical parameters with OS and DFS. A *p*-value of <0.05 was considered to be statistically significant.

## 5. Conclusions

For MSI-H GC, EMAST+ tumors showed a more favorable prognosis and were associated with a higher frequency of several DNA-repair-associated genetic mutations than EMAST− tumors. EMAST+/MSI-H tumors are likely to be a different entity from EMAST−/MSI-H tumors. Combined EMAST/MSI status is recommended for the evaluation of immunotherapy for GC treatment. PI3K/AKT pathway mutations are more frequent in EMAST+ and/or MSI-H tumors than in EMAST−/MSS tumors. Further in vivo and in vitro studies are required to investigate of the correlation of EMAST/MSI status and genetic mutations in DNA-repair-associated genes and the PI3K/AKT pathway in GC. We hope our results can shed light on GC treatment, including immunotherapy and even targeted therapy.

## Figures and Tables

**Table 1:** Clinical profiles among patients according to the EMAST and MSI status.

Clinical Profiles	EMAST Status			MSI Status		
Variables	− n = 284 n (%)	+ n = 76 n (%)	*p*-Value	MSS n = 301 n (%)	MSI-H n = 59 n (%)	*p*-Value
Age (y/o)			0.327			**0.028**
<45	20 (7.0)	3 (3.9)		23 (7.6)	0	
≥45	264 (93.0)	73 (96.1)		278 (92.4)	59 (100)	
Gender (M/F)	189/95	56/20	0.236	204/97	41/18	0.796
Tumor size (<5/≥5 cm)	98/186	21/55	0.258	107/194	12/47	**0.023**
Cell differentiation			0.929			0.559
Poor	162 (57.0)	43 (56.6)		174 (57.8)	31 (52.5)	
Moderate	119 (41.9)	44 (43.4)		124 (41.2)	28 (47.5)	
Well	3 (1.1)	0		3 (1.0)	0	
Gross appearance			**0.005**			0.510
Superficial type	18 (6.3)	8 (10.5)		24 (8.0)	2 (3.4)	
Borrmann type 1 and 2	74 (26.1)	32 (42.1)		87 (28.9)	19 (32.2)	
Borrmann type 3 and 4	192 (67.6)	36 (47.4)		190 (63.1)	38 (64.4)	
Lymphovascular invasion	201 (70.8)	52 (68.4)	0.690	208 (69.1)	45 (76.3)	0.271
Lymphoid stroma	27 (9.5)	12 (15.8)	0.118	30 (10.0)	9 (15.3)	0.232
EBV infection	33 (11.6)	12 (15.8)	0.329	33 (11.0)	12 (20.3)	**0.046**
HP infection	104 (36.6)	11 (14.5)	**<0.001**	105 (34.9)	10 (16.9)	**0.007**
*PIK3CA* amplification	76 (26.8)	16 (21.1)	0.311	78 (25.9)	14 (23.7)	0.725
Pathological T category			**0.049**			0.232
T1	32 (11.3)	11 (14.5)		37 (12.3)	6 (10.2)	
T2	30 (10.6)	16 (21.1)		34 (11.3)	12 (20.3)	
T3	125 (44.0)	31 (40.8)		130 (43.2)	26 (44.1)	
T4	97 (34.2)	18 (23.7)		100 (33.2)	15 (25.4)	
Pathological N category			0.121			0.911
N0	65 (22.9)	26 (34.2)		77 (25.6)	14 (23.7)	
N1	42 (14.8)	14 (18.4)		45 (15.0)	11 (18.6)	
N2	53 (18.7)	11 (14.5)		54 (17.9)	10 (16.9)	
N3	124 (43.7)	25 (32.9)		125 (41.5)	24 (40.7)	
Pathological TNM Stage			0.050			0.507
I	40 (14.1)	20 (26.3)		48 (15.9)	12 (20.3)	
II	68 (23.9)	17 (22.4)		75 (24.9)	10 (16.9)	
III	113 (48.9)	34 (44.7)		142 (47.2)	31 (52.5)	
IV	37 (13.0)	5 (6.6)		36 (12.0)	6 (10.2)	
Genetic mutation						
*PI3K/AKT* pathway	22 (7.7)	19 (25.0)	**<0.001**	25 (8.3)	16 (27.1)	**<0.001**
*ARID1A*	11 (3.9)	11 (14.5)	**0.001**	18 (6.0)	4 (6.8)	0.815
*TP53*	27 (9.5)	5 (6.6)	0.426	30 (10.0)	2 (3.4)	0.105
*KRAS*	8 (2.8)	3 (3.9)	0.611	7 (2.3)	4 (6.8)	0.069
*BRAF*	0	1 (1.3)	0.053	1 (0.3)	0	0.658

EMAST: elevated microsatellite alterations at selected tetranucleotide repeats; MSI-H: microsatellite instability-high; MSS: microsatellite stable; EBV: Epstein–Barr virus; HP: Helicobacter pylori. Bold: statistically significant

**Table 2:** Clinical profiles among patients according to the EMAST/MSI status.

Clinical Profiles	EMAST/MSI Status				
Variables	+/MSI-H n = 35 n (%)	+/MSS n = 41 n (%)	−/MSI-H n = 24 n (%)	−/MSS n =2 60 n (%)	*p*-Value
Age					0.117
<45 years	0	3 (7.3)	0	20 (7.7)	
≥45 years	35 (100)	38 (92.7)	24 (100)	240 (92.3)	
Gender (M/F)	25/10	31/10	16/8	173/87	0.669
Tumor size (<5/≥5 cm)	8/27	13/28	4/20	94/166	0.126
Cell differentiation					0.889
Poor	23 (65.7)	20 (48.8)	8 (33.3)	154 (59.2)	
Moderate	12 (34.3)	21 (51.2)	16 (66.7)	103 (39.6)	
Well	0	0	0	3 (1.2)	
Gross appearance					**0.030**
Superficial type	2 (5.7)	6 (14.6)	0	18 (6.9)	
Borrmann type 1 and 2	17 (48.6)	15 (36.6)	2 (8.3)	72 (27.7)	
Borrmann type 3 and 4	16 (45.7)	20 (48.8)	22 (91.7)	170 (65.4)	
Lymphovascular invasion	23 (65.7)	29 (70.7)	22 (91.7)	179 (68.8)	0.118
Lymphoid stroma	9 (25.7)	3 (7.3)	0	27 (10.4)	0.094
EBV infection	6 (17.1)	6 (14.6)	6 (25.0)	27 (10.4)	0.197
HP infection	6 (17.1)	5 (12.2)	4 (16.7)	100 (38.5)	**<0.001**
*PIK3CA* amplification	6 (17.1)	10 (24.4)	8 (33.3)	68 (26.2)	0.543
Pathological T category					**0.037**
T1	6 (17.1)	5 (12.2)	0	32 (12.3)	
T2	8 (22.9)	8 (19.5)	4 (16.7)	26 (10.0)	
T3	14 (40.0)	17 (41.5)	12 (50.0)	113 (43.5)	
T4	7 (20.0)	11 (26.8)	8 (33.3)	89 (34.2)	
Pathological N category					0.062
N0	12 (34.3)	14 (34.1)	2 (8.3)	63 (24.2)	
N1	7 (20.0)	7 (17.1)	4 (16.7)	38 (14.6)	
N2	6 (17.1)	5 (12.2)	4 (16.7)	49 (18.8)	
N3	10 (28.6)	15 (36.6)	14 (58.3)	110 (42.3)	
Pathological TNM Stage					**0.038**
I	10 (28.6)	10 (24.4)	2 (8.3)	38 (14.6)	
II	8 (22.9)	9 (22.0)	2 (8.3)	66 (25.4)	
III	15 (42.9)	19 (46.3)	16 (66.7)	123 (47.3)	
IV	2 (5.7)	3 (7.3)	4 (16.7)	33 (12.7)	
Genetic mutation					
*PI3K/AKT* pathway	8 (22.9)	11 (26.8)	8 (33.3)	14 (5.4)	**<0.001**
*ARID1A*	2 (5.7)	9 (22.0)	2 (8.3)	9 (3.5)	**0.002**
*TP53*	0	5 (12.2)	2 (8.3)	25 (9.6)	0.066
*KRAS*	2 (5.7)	1 (2.4)	2 (8.3)	6 (2.3)	0.422
*BRAF*	0	1	0	0	0.224

EMAST: elevated microsatellite alterations at selected tetranucleotide repeats; MSI-H: microsatellite instability-high; MSS: microsatellite stable; EBV: Epstein–Barr virus; HP: Helicobacter pylori. Bold: statistically significant.

**Table 3:** Genetic mutations using NGS method, according to the EMAST and MSI status.

	EMAST Status			MSI Status		
Genes	− n = 76	+ n = 74	*p*-Value	MSS n = 92	MSI-H n = 58	*p*-Value
EXO1	2 (2.6)	9 (12.2)	**0.025**	1 (1.1)	10 (17.2)	**<0.001**
EPCAM	0	4 (5.4)	**0.040**	0	4 (6.9)	**0.011**
MSH2	0	17 (23.0)	**<0.001**	9 (9.8)	8 (13.8)	0.451
MSH6	6 (7.9)	12 (16.2)	0.117	8 (8.7)	10 (17.2)	0.117
PCNA	0	0	-	0	0	-
PMS1	2 (2.6)	2 (2.7)	0.978	0	4 (6.9)	**0.011**
PMS2	2 (2.6)	1 (1.4)	0.576	3 (3.3)	0	0.165
TGFBR2	5 (6.6)	15 (20.3)	**0.014**	8 (8.7)	12 (20.7)	**0.035**
MLH1	0	4 (5.4)	**0.040**	4 (4.3)	0	0.107
CTNNB1	3 (3.9)	4 (5.4)	0.672	7 (7.6)	0	**0.031**
MSH3	4 (5.3)	19 (25.7)	**0.001**	13 (14.1)	10 (17.2)	0.607
POLE	2 (2.6)	11 (14.9)	**0.008**	9 (9.8)	4 (6.9)	0.541
AXIN1	1 (1.3)	11 (14.9)	**0.002**	6 (6.5)	6 (10.3)	0.401
AXIN2	0	4 (5.4)	**0.040**	4 (4.3)	0	0.107
BAX	3 (3.9)	15 (20.3)	**0.002**	6 (6.5)	12 (20.7)	**0.009**
POLD1	3 (3.9)	9 (12.2)	0.064	6 (6.5)	6 (10.3)	0.401

EMAST: elevated microsatellite alterations at selected tetranucleotide repeats; MSI-H: microsatellite instability-high; MSS: microsatellite stable. Bold: statistically significant.

**Table 4:** Genetic mutations using NGS method, according to the EMAST/MSI status.

	EMAST/MSI Status				*p*-Value
Genes	+/MSI-H (n = 34)	+/MSS (n = 40)	−/MSI-H (n = 24)	−/MSS (n = 52)	
EXO1	8 (23.5)	1 (2.5)	2 (8.3)	0	**0.001**
EPCAM	4 (11.8)	0	0	0	**0.005**
MSH2	8 (23.5)	9 (22.5)	0	0	**<0.001**
MSH6	8 (23.5)	4 (10.0)	2 (8.3)	4 (7.7)	**0.048**
PCNA	0	0	0	0	-
PMS1	2 (5.9)	0	2 (8.3)	0	0.281
PMS2	0	1 (2.5)	0	2 (3.8)	0.294
TGFBR2	10 (29.4)	5 (12.5)	2 (8.3)	3 (5.8)	**0.003**
MLH1	0	4 (10.0)	0	0	0.281
CTNNB1	0	4 (10.0)	0	3 (5.8)	0.596
MSH3	8 (23.5)	11 (27.5)	2 (8.3)	2 (3.8)	**0.002**
POLE	4 (11.8)	7 (17.5)	0	2 (3.8)	**0.045**
AXIN1	6 (17.6)	5 (12.5)	0	1 (1.9)	**0.003**
AXIN2	0	4 (10.0)	0	0	0.281
BAX	10 (29.4)	5 (12.5)	2 (8.3)	1 (1.9)	**<0.001**
POLD1	6 (17.6)	3 (7.5)	0	3 (5.8)	0.055

Statistically significant; EMAST: elevated microsatellite alterations at selected tetranucleotide repeats; MSI-H: microsatellite instability-high; MSS: microsatellite stable. Bold: statistically significant.

**Table 5:** The patterns of initial recurrence of gastric cancer after curative surgery, according to EMAST and MSI status.

Recurrence Patterns	EMAST (−) n = 217	EMAST (+) n = 58	*p*-Value	MSS n = 230	MSI-H n = 45	*p*-Value
Total recurrence	60 (27.6)	9 (15.5)	0.058	61 (26.5)	8 (17.8)	0.216
Locoregional recurrence	13 (6.0)	4 (6.9)	0.763	15 (6.5)	2 (4.4)	0.597
Distant metastasis	45 (20.7)	5 (8.6)	**0.034**	44 (19.1)	6 (13.3)	0.356
Peritoneal dissemination	20 (9.2)	1 (1.7)	0.090	17 (7.4)	4 (8.9)	0.759
Hematogenous metastasis	23 (10.6)	3 (5.2)	0.210	22 (9.6)	4 (8.9)	0.887
Liver	18 (8.3)	2 (3.4)		16 (7.0)	4 (8.9)	
Lung	1 (0.5)	2 (3.4)		3 (1.3)	0	
Bone	3 (1.4)	0		3 (1.3)	0	
Skin	1 (0.5)	0		1 (0.4)	0	
Distant lymphatic recurrence	12 (5.5)	1 (1.7)	0.313	13 (5.7)	0	0.136
Virchow’s node	1 (0.5)	0		1 (0.4)	0	
Para-aortic lymph node	12 (5.7)	1 (2.3)		13 (5.7)	0	

Some patients had more than one initial recurrence pattern; EMAST: elevated microsatellite alterations at selected tetranucleotide repeats; MSI-H: microsatellite instability-high; MSS: microsatellite stable. Bold: statistically significant.

**Table 6:** The patterns of initial recurrence of gastric cancer after curative surgery, according to EMAST status.

Recurrence Patterns	EMAST/MSI Status				
	+/MSI-H n = 29	+/MSS n = 29	−/MSI-H n = 16	−/MSS n = 201	*p*-Value
Total recurrence	0	9 (31.0)	8 (50.0)	52 (25.9)	**0.001**
Locoregional recurrence	0	4 (13.8)	2 (12.5)	11 (5.5)	0.991
Distant metastasis	0	5 (17.2)	6 (37.5)	39 (19.4)	**0.014**
Peritoneal dissemination	0	1 (3.4)	4 (25.0)	16 (8.0)	**0.019**
Hematogenous metastasis	0	3 (10.3)	4 (25.0)	19 (9.5)	0.056
Liver	0	2 (3.4)	4 (25.0)	14 (7.0)	
Lung	0	2 (6.9)	0	1 (0.5)	
Bone	0	0	0	3 (1.5)	
Skin	0	0	0	1 (0.5)	
Distant lymphatic recurrence	0	1 (3.4)	0	12 (6.0)	0.127
Virchow’s node	0	0	0	1 (0.5)	
Para-aortic lymph node	0	1 (3.4)	0	12 (6.0)	

Some patients had more than one initial recurrence pattern; EMAST: elevated microsatellite alterations at selected tetranucleotide repeats; MSI-H: microsatellite instability-high; MSS: microsatellite stable. Bold: statistically significant.

**Table 7:** Multivariate Cox proportional-hazards model for the analysis of the overall survival and disease-free survival for gastric cancer patients after curative surgery.

Risk Factors	Overall Survival			Disease-free Survival		
	HR	95% CI	*p*-Value	HR	95% CI	*p*-Value
Age (y/o)			0.204			0.305
<45	1.00			1.00		
≥45	1.66	0.759–3.616		1.47	0.705–3.054	
Gender			0.068			0.206
M	1.00			1.00		
F	0.67	0.435–1.030		0.77	0.505–1.159	
Tumor size (cm)			0.590			0.622
<5	1.00			1.00		
≥5	1.11	0.755–1.638		1.10	0.755–1.601	
Lymphovascular invasion			**0.046**			**0.045**
Absent	1.00			1.00		
Present	1.58	1.009–2.487		1.57	1.010–2.433	
Lauren’s classification			**0.022**			**0.033**
Intestinal type	1.00			1.00		
Diffuse type	1.55	1.064–2.268		1.50	1.032–2.169	
Pathological TNM stage			**0.001**			**0.002**
I	1.00			1.00		
II	1.02	0.555–1.867		1.05	0.581–1.895	
III	2.18	1.184–4.011		2.18	1.208–3.935	
EMAST status			0.384			0.490
−	1.00			1.00		
+	1.22	0.781–1.900		1.17	0.748–1.831	
MSI status			0.854			0.986
MSI-H	1.00			1.00		
MSS	1.05	0.627–1.758		1.01	0.598–1.688	

EMAST: elevated microsatellite alterations at selected tetranucleotide repeats; MSI: microsatellite instability; MSI-H: microsatellite instability-high; MSS: microsatellite stable. Bold: statistically significant.
